# Decellularized and Engineered Tendons as Biological Substitutes: A Critical Review

**DOI:** 10.1155/2016/7276150

**Published:** 2016-01-06

**Authors:** Arianna B. Lovati, Marta Bottagisio, Matteo Moretti

**Affiliations:** Cell and Tissue Engineering Laboratory, IRCCS Galeazzi Orthopaedic Institute, Via R. Galeazzi 4, 20161 Milan, Italy

## Abstract

Tendon ruptures are a great burden in clinics. Finding a proper graft material as a substitute for tendon repair is one of the main challenges in orthopaedics, for which the requirement of a biological scaffold would be different for each clinical application. Among biological scaffolds, the use of decellularized tendon-derived matrix increasingly represents an interesting approach to treat tendon ruptures. We analyzed* in vitro* and* in vivo* studies focused on the development of efficient protocols for the decellularization and for the cell reseeding of the tendon matrix to obtain medical devices for tendon substitution. Our review considered also the proper tendon source and preclinical animal models with the aim of entering into clinical trials. The results highlight a wide panorama in terms of allogenic or xenogeneic tendon sources, specimen dimensions, physical or chemical decellularization techniques, and the cell type variety for reseeding from terminally differentiated to undifferentiated mesenchymal stem cells and their static or dynamic culture employed to generate implantable constructs tested in different animal models. We try to identify the most efficient approach to achieve an optimal biological scaffold for biomechanics and intrinsic properties, resembling the native tendon and being applicable in clinics in the near future, with particular attention to the Achilles tendon substitution.

## 1. Introduction

Tendon ruptures, frequently associated with tendinopathy, and both tendon retractions and extensive loss after trauma represent a great burden in surgical reconstruction. The most commonly affected tendons are the finger and hand flexors and extensors [1, 2], the rotator cuff [3], and the Achilles tendon [4]. In particular, acute Achilles tendon ruptures have an increasing incidence of 18 per 100,000 [5]. Overall, these injuries are directly combined with high health and socioeconomic costs and long-term postoperative rehabilitation and indirectly with the loss of productivity.

Tendon repair is a slow process in order to reestablish the tendon fiber continuity and the functional physiological mechanism. Their poor ability in healing is commonly due to the low cell density (5%), scarce oxygen and nutrient supply, and abnormal collagen deposition [6] that together lead to scar tissue formation and adhesion, thereby impairing the normal tissue function. Indeed, the functionality of tendon biomechanics is primarily related to their complex matrix architecture. According to these premises, finding a suitable graft material as a substitute for tendon reconstruction is one of the main challenges in orthopaedics. To reproduce the biomechanical and biochemical properties similar to the native tissue structure, a tendon substitute should have specific biological properties, including biocompatibility, absence of inflammatory or immune response, and a close interaction with tendon cells, as well as adequate mechanical properties [7]. Wide tendon damage needs to be repaired by large amounts of synthetic or biological tissue substitutes. In view of this, novel approaches for tendon substitution have been recently proposed and marketed for clinical use, including synthetic biomaterials or biological grafts [8]. Nevertheless, synthetic materials for tendon defects have provided poor results with regard to the healing and mechanical properties [7]. Thus, biological grafts, including auto-, allo-, and xenografts, have been widely investigated to satisfy the aforementioned required features for an adequate tendon repair, and, nowadays, they represent the gold standard for tendon repair [7]. Autografts are of limited availability of the dimension and sites of harvest, with high donor site morbidity and prolonged surgical time [9]. Allografts from cadavers are at higher risk of disease transmission and might induce a chronic immune response requiring immunosuppressive approaches [9]. During the last twenty years, decellularization protocols of allografts and xenografts have been investigated to remove the cell-related immunogenicity by preserving the integrity of collagen structure and the biological characteristics of the tendon matrix. In particular, the use of animal-derived xenografts offers great amounts of collectable tissue with the possibility to produce commercial scaffolds from different collagen structures [7, 8].

Several decellularization methods, including physical, chemical, and enzymatic ones and combinations of these techniques, have been described [10]. Most of the published studies describe the production of decellularized tissue scaffolds derived from nonhomologous anatomical sites (e.g., intestinal submucosa, dermal patch, and pericardium). However, decellularized matrix derived from site-specific homologous tissue may be more suitable than a non-site-specific source, in particular when referring to musculoskeletal tissues due to their structural and biomechanical properties [11]. In fact, homologous sources better provide a site-specific extracellular matrix (ECM) offering a valid scaffold to host cell ingrowth and to highly respond to tensile loading typical of the native tendon [9]. While the tendon is a complex three-dimensional structure consisting of well-organized collagen fibers, the tissue-specific decellularized matrix may provide a suitable and natural scaffold with the same native orientation of collagen fibers that has not been synthetically created in the laboratory yet [9]. The current challenge is to develop xenogeneic biological matrices that offer apparent advantages over both synthetic and human-derived scaffolds in view of minimal morbidity and suitable mechanical properties.

This review provides an overview of* in vitro* and* in vivo* studies on decellularization procedures of tendon tissue derived from animals or humans. In particular, we analyzed the efficacy of decellularization techniques followed or not by cell reseeding to identify the most suitable approach to obtain a functional natural tendon substitute that can be translated from preclinical models to clinics for tendon replacement.

## 2. Methods: Inclusion Criteria

**Figure 1:**
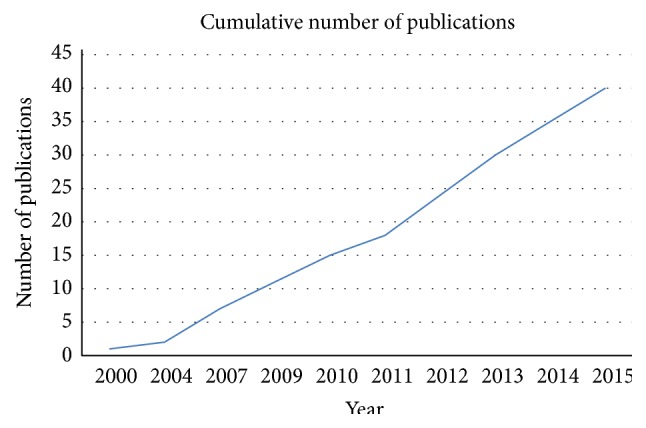
Cumulative number of publications over years. Publication trend from January 2000 until April 2015 on studies performing both* in vitro* and* in vivo* tendon tissue decellularization.

**Figure 2:**
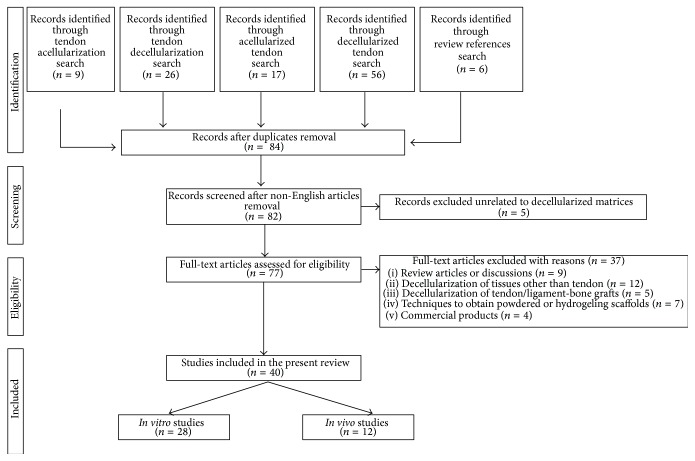
Research strategy. Flow chart of selection process.

The literature search was performed in PubMed database, by considering articles published in English from 2000 until April 2015, as depicted in Figure 1. The search strategy was conducted by searching “decellularization, decellularized, acellularization, acellularized” combined with the keyword “tendon” (Figure 2). Review articles were used to complete our study by including publications that were not present on the PubMed database according to our searching criteria. Our search was focalized both on decellularization protocols in order to obtain a pure matrix from tendon tissue eventually reseeded with a cell source and on the functionality of the biological scaffold after* in vivo* implantation. With this strategy, we found 77 studies. Twelve studies were excluded because of concerning the decellularization of tissue other than tendons. Of the remaining sixty-five studies, sixteen studies were excluded because of concerning decellularization of tendon-bone grafts, tendon decellularization until matrix powdering or hydrogeling, and commercial products of which the decellularization techniques are not described. Finally, five studies were not included because of describing allografts,* in vitro* cell culture unrelated to decellularized matrix, and bioactive sutures related to tendon repair but not with tendon substitution or augmentation. Finally, review articles were also excluded. In conclusion, a total of 40 studies, 28* in vitro* and 12* in vivo*, were included in this review of the literature. The review results are reported in Tables 1 and 2 for the* in vitro* and* in vivo* published studies, respectively. To give a global overview of the most employed reagents, cell sources, and construct analyses, we present pie charts in Section 3. The pie chart percentages were calculated by considering the number of papers employing the aforementioned parameters.

## 3. Results and Discussion

### 3.1. Tendon Sources and Specimen Dimensions

**Figure 3:**
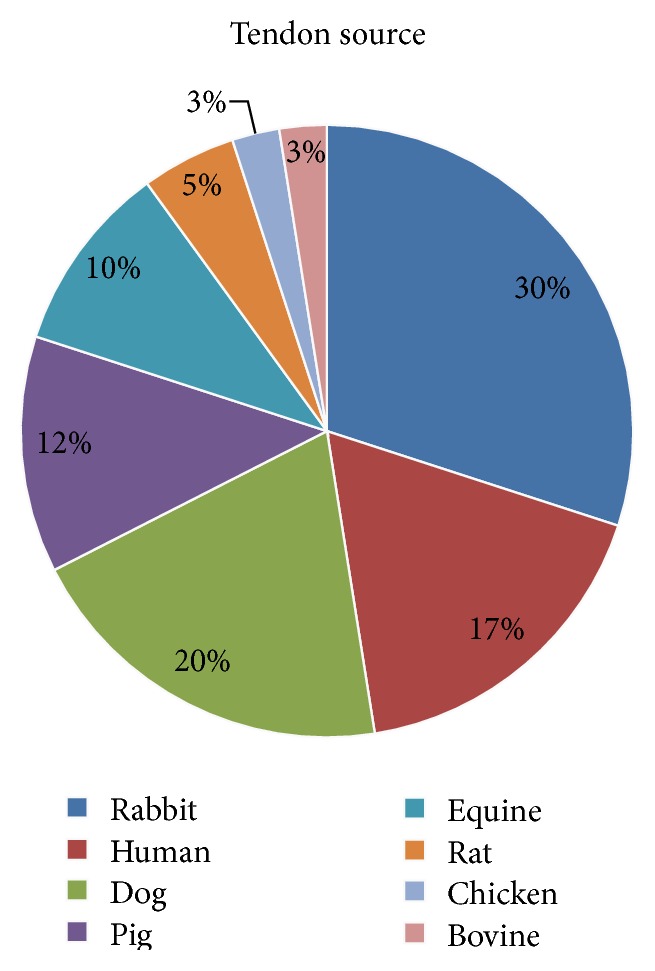
Tendon sources. The pie chart shows the relative distribution of species from which tendons have been harvested to be decellularized.

In this review, we considered studies that have used tendons derived from different mammalians as biological scaffolds. Most of the analyzed articles described decellularization protocols on flexor, patellar, or Achilles tendons harvested from rabbits [11–22], rats [23, 24], or evolutionary less developed species like chicken [25], before of human  [26–32], or other animal tissues such as from canine [33–39], porcine [40–44], equine [45–48], and bovine tendons [49].  Figure 3 displays the distribution of tendon sources from different species. In most cases, the dimensions of the native tendon tissue for the decellularization are reported as surface area ranging from 0.45 to 12 cm^2^ (mean 3.21 ± 3.24 cm^2^), tissue length ranging from 1 to 8 cm (mean 3.25 ± 1.80 cm), or tissue thickness with values ranging from 0.08 to 5 mm (mean 1.16 ± 1.40 mm). Some authors left undefined the scaffold sizes [15, 16, 24, 33, 42] or thickness [11, 12, 17, 18, 21–24, 26–32, 34, 41]. Many of these studies are not intended to produce a scaffold for human use. Despite this, the production of a scaffold of adequate dimensions for tendon replacement in humans is considered challenging and relevant for clinical application, particularly regarding the Achilles tendon reconstruction.

#### 3.1.1. Observation on Tendon Sources and Dimensions

The clinical use of decellularized biological scaffolds imperatively requires any host adverse responses, safety, proper dimensions, and adequate availability. Importantly, the ultimate species—providing organs for xenotransplantation—should consider anatomical-physiological and ethical concerns, as well as accessibility related to breeding and slaughtering. Studies using tendons from humans [26–32] should be considered cautiously because of the aforementioned problems such as limited availability from cadavers and risks of disease transmission. Furthermore, the quality and structural organization of the tendon matrix could be influenced by donor age, anatomical site, and loading history [50]; thus the quality control of the source material might play an important role in the success of the graft. Tendons derived from inferior species, including chicken [25], rat [23, 24], and rabbit [11–22], have limited dimensions and amounts to be used as clinical substitutes, though these animals are useful to test the efficacy of decellularization protocols. Moreover, according to the common slaughter procedures for chicken and rabbits, the tendon harvesting is not possible due to the lack of slaughter waste products. Thus, dedicated subjects need to be scarified for this purpose with correlated ethical issues. Canine tendons have appropriate dimensions, yet the use of dog [33–39] native tissues on a large scale is not practical and could be also subjected to ethical issues. For these aforementioned reasons, the large availability in terms of quantity and dimensions of tendon sources from commonly slaughtered animals as pigs [40–44], equine [45–48], and bovine [49] could be a valid choice. Despite the apparent advantages in using pigs as donor species, it retains a great variety of transmittable pathogens to humans [51]. According to this, equine xenografts are becoming even more used for reconstructive surgery [7, 8], due to a significantly inferior presence and often geographically limited zoonotic diseases [52].

Among several types of tendon in humans that need to be repaired, the Achilles tendon is one of the largest collagen structures that undergoes severe injuries. Moreover, it is also the most studied tendon in terms of biomechanical and structural properties [53–56]. Thus, the aforementioned features make the Achilles tendon a challenging structure to be replaced, and it could be considered as a benchmark in generating a tendon substitute. Thus, obtaining a performing decellularized tendon matrix for its substitution requires big efforts to get close to the native dimensions, preserving the functional biomechanical and structural properties. Most of the studies included in this review focused on decellularization processes of small tendon portions because they were aimed at optimizing the decellularization protocols. Although these approaches led to interesting results in terms of cell component removal, they could not be necessarily adequate for greater-sized specimens. Only a few studies worked with suitable tendon dimensions [28, 41, 43] to be used in clinics. The increased resistance of decellularization detergent penetration or of cell colonization during recellularization represents two limitations in processing large tendon specimens. These problems were widely described by authors who performed static approaches for both the decellularization and the reseeding processes with particular reference to the thickness of the tissue [25, 43]. To overcome these hindrances, two studies carried out innovative strategies such as dynamic techniques [41], tissue surface scoring, or superficial cutting [28]. Specifically, Lee et al. [41] described tension and torsion stimuli to improve the detergent penetration, ameliorating also the biomechanical properties of the decellularized matrix. With the same purpose, Woon et al. [28] proposed surface scoring of the tendon to create a breach for both the cell penetration and the attachment, demonstrating the treatment effectiveness. However, all these techniques present some limitations in terms of weakening the decellularized tendon matrix.

### 3.2. The Panorama of Decellularization Protocols and Cell Reseeding Strategies for Tendon Tissue

**Figure 4:**
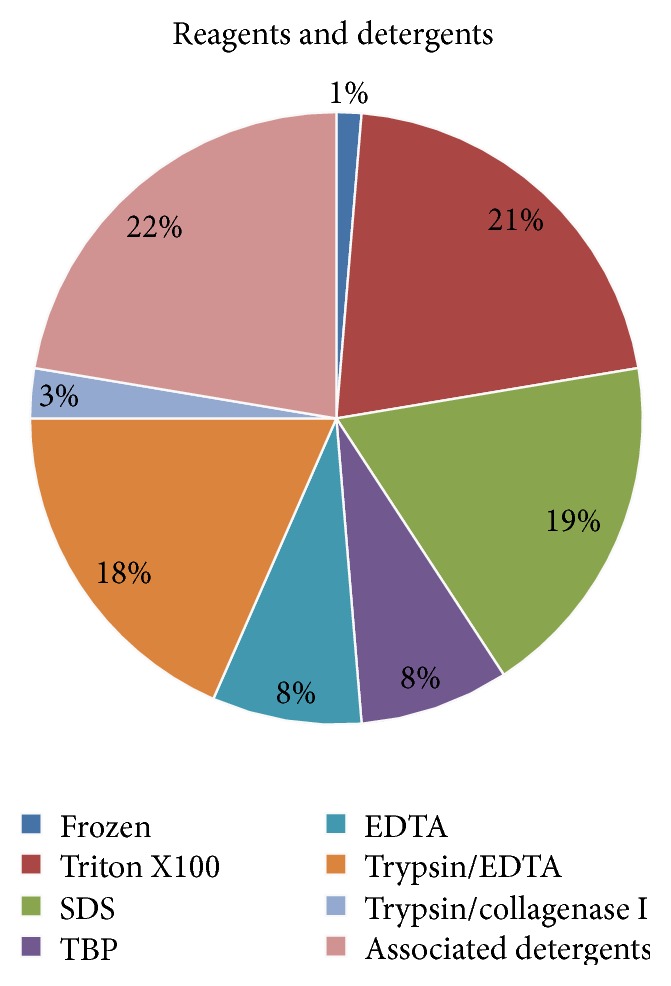
Reagents and detergents. The pie chart shows the percentage of physical, chemical, enzymatic, or associated detergents employed for tendon decellularization.

Several physical or chemical treatments have been described for tendon decellularization to obtain an antigen-free ECM that could support biomechanical loading similar to the native healthy tendons. Physical treatments are the most commonly used to interrupt the binding of resident cells to the collagen matrix and to disrupt the cell membrane. These treatments include mechanical forces, like ultrasonication [43], and repeated freeze-thaw cycles at −20/80°C [10, 11, 13–21, 26, 27, 29–39, 44–46, 48]. These latest treatments are the most frequently used and can be associated with or followed by freeze-thaw cycles in liquid nitrogen [20, 35, 36, 39, 44, 45, 57]. Chemical treatments can directly start in fresh samples without any previous cycles of freezing and thawing [11, 13, 25, 26, 29, 42, 43, 45] and are commonly used to degrade cell components, remains, and antigens. In particular, chemicals act on cell cytoplasm, nuclear membrane, and lipid/protein interactions. Nonionic (Triton X100) [11–14, 17, 18, 21–24, 31, 33, 34, 40, 42, 45], ionic (Sodium Dodecyl Sulfate, SDS) [11, 13, 15, 16, 19, 23, 26, 31, 42, 43, 45, 46, 48, 49], and zwitterionic (Tributyl Phosphate, TBP) [13, 19, 23, 31, 42, 46] detergents are the most employed agents to obtain a complete tendon decellularization. These reagents can be used either alone or in combination to increase their effect [11, 13, 23, 42, 46]. Hypotonic and hypertonic solutions or EDTA buffers are frequently used with physical [13, 27, 28, 31, 32, 49] or chemical treatments in order to better lysate cells [27–30, 32, 40]. EDTA can also be coupled with specific inhibitors (aprotinin and leupeptin) to reduce the protease release that damages the ECM structure [26, 43]. Because tendons have a well-organized, compact structure, the collagen matrix permeability to detergents and reseeded cells needs to be improved by means of acid solutions (e.g., peracetic, acetic, hydrochloridric, and sulfuric acids) [25, 27–30, 32, 42] or by scoring or perforating the tendon surface with multiple slits [28, 34]. Moreover, in order to allow extrinsic cells to migrate into the acellular scaffold, some authors performed various intensities of ultrasonication with the aim of producing a microscopically more open porous matrix without damaging the overall architecture of the scaffold [43]. Frequently, enzymatic treatments were associated with physical and chemical protocols. Enzymatic substances like Trypsin were frequently combined with EDTA [11, 14, 17, 18, 21, 22, 24, 25, 33, 34, 40, 46, 48], with collagenase type I [12, 41], or with chemical treatments [11] to disrupt peptide bonds at 37°C. Importantly, in order to avoid a host immune response, endonucleases (RNAse and DNAse) are commonly used to complete the decellularization process cleaving the RNA and DNA remains [13, 25, 26, 35–39, 43, 44, 46, 48, 57]. Only 27.5% of the analyzed studies carried out a comparison among different decellularization protocols allowing a deeper insight into the efficacy of chemical treatments [11, 13, 19, 23, 27, 31, 40, 42, 43, 45, 46]. In Figure 4, the most frequently used reagents and detergents are displayed for a better comprehension.

**Figure 5:**
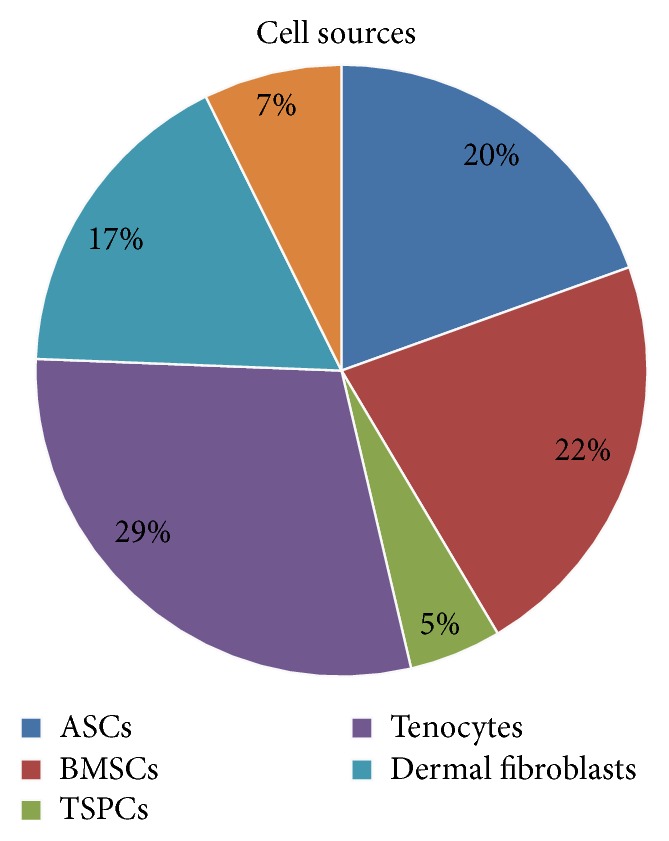
Cell sources. The pie chart shows the relative distribution of cell sources used to reseed the decellularized matrix.

About 80% of studies analyzed in this review performed cell reseeding of the decellularized tendon tissue using different kinds of cells belonging to the mesenchymal lineage (bone-marrow- [21, 34–36, 39, 46–48, 57], adipose- [17, 18, 21, 26, 28, 30, 32, 45], or tendon-derived stromal cells [44, 57]) or the fibroblastic lineage (tenocytes [11, 12, 14, 18, 20–22, 24, 30, 40, 43, 47] and dermal [15, 16, 19, 27, 30, 31, 37] and tendon sheath fibroblasts [11, 17, 21]), as depicted in Figure 5. The number of cells used for reseeding was differently reported as cells/cm^2^ or cells/mL and varied from 2 × 10^4^ to 4 × 10^6^ (mean 8 × 10^5^ ± 1.3 × 10^6^) and from 1 × 10^5^ to 2 × 10^7^ (mean 2.6 × 10^6^ ± 4 × 10^6^), respectively. Cell reseeding was performed through the static (72%) or dynamic cultures (28%) using simple rotating [16, 21, 26, 27, 29, 30, 39] and cyclic strain cultures [39, 48] or specific commercial bioreactors [14, 17] for a culture time ranging from 24 hours to 8 days (mean 10 ± 11.6 days).

#### 3.2.1. Investigations to Assess the Decellularization and Recellularization of Tendon Matrix

**Figure 6:**
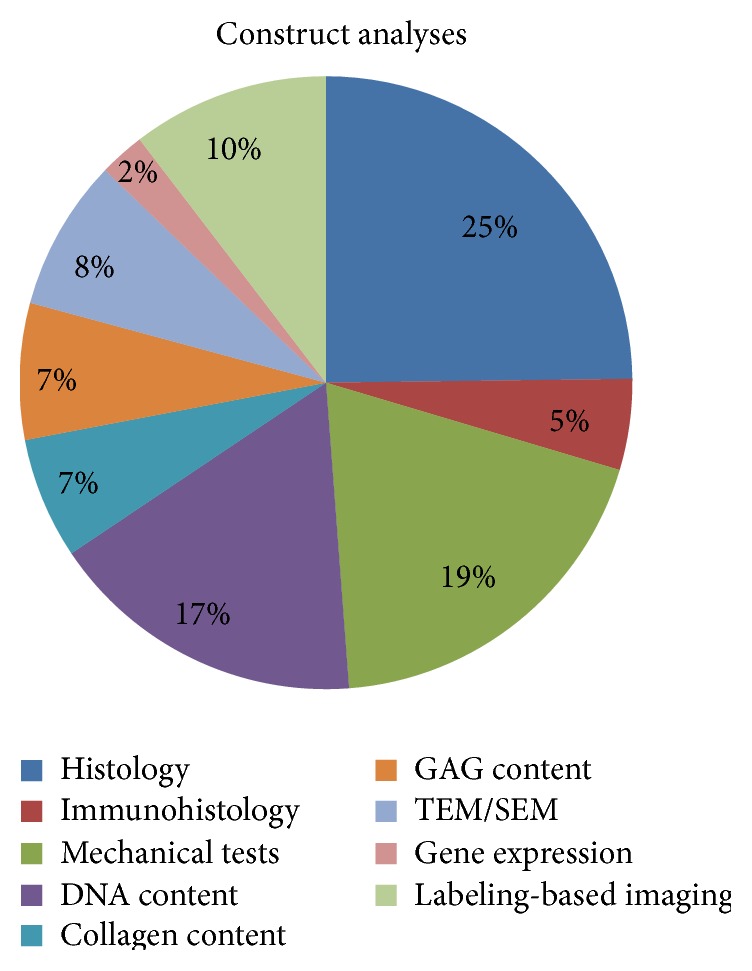
Construct analyses. The pie chart shows the relative distribution of analyses performed to assess the quality of the decellularization and reseeding of the tendon matrix.

Specific analytical investigations are normally performed to verify the efficacy of the decellularization protocols or the capability of the decellularized tissues to be efficiently colonized by cells. Among various analyses that were commonly performed either* in vitro* or after* in vivo* implantation, the most employed techniques to verify the intrinsic and biomechanical qualities of the construct were represented by histology (Haematoxylin and Eosin [11–14, 16–19, 21–29, 31–51], Masson's Trichrome [26, 44, 46], Alcian Blue [26, 40], Prussian Blue [45], Nuclear Fast Red [40], SYTO Green [17, 27, 28, 30–32], and DAPI [24, 25, 37, 40, 41, 43, 45]) and mechanical testing (ultimate tensile strength and elastic modulus [12–14, 17, 18, 20, 23, 27, 28, 31–34, 38, 39, 42, 43, 46, 48, 49, 57], stiffness [16, 19, 25, 29, 35, 37, 41, 44], and elongation [16]), respectively. Furthermore, to detect the presence of residual cells in decellularized tissues or reseeded constructs, DNA content [12, 14, 17, 19, 25–28, 30–32, 34, 37, 40, 41, 44–48, 57] was performed as principal investigation together with other interesting analyses, such as electron microscopy (SEM [25, 33, 37, 39, 41, 42, 44, 46, 49, 57] and TEM [28, 44, 45]) and labeling-based imaging to discriminate viable cells [11, 20, 27, 28, 31, 32, 34–36, 39, 40, 42, 43, 45, 46, 57]. Proteoglycans and collagen fibers are the principal components of the tendon ECM and their conservation within a decellularized/recellularized scaffold is mandatory. For this reason, biochemical assays [19, 27, 31, 37, 40–44, 46–48] or gene expression analyses [35, 36, 44, 47] (collagen types I and III, tenomodulin, scleraxis, and cartilage oligomeric matrix protein), frequently associated with immunohistochemical investigations [20, 26, 28, 30, 32, 43], have been performed to analyze their content within the treated constructs. Animal models have been used to define the inflammatory and immune response upon scaffold implantation through specific immunohistochemical analyses [29], as well as through the detection of inflammatory matrix metalloproteinases (MMP2, MMP3, and MMP13) [35, 36]. Only one study analyzed directly* in vivo* the construct integration within native tissue after implantation by using the magnetic resonance [38]. In Figure 6, the distribution of typical analyses of decellularized/recellularized constructs is resumed.

#### 3.2.2. Observation on Decellularization and Reseeding Protocols

The ultimate goal of a decellularization process is to obtain a scaffold wholly free of cellular components, thus avoiding any host immune reaction. In addition, the removal of remaining reagents is mandatory to contain the inflammatory response after transplantation. Thus, one of the most useful and employed approaches to contain the inflammatory reaction to biological scaffolds is the use of endonucleases (DNAse and RNAse) able to remove potential cell remaining from the decellularized matrix. Anyway, with the aim of controlling these adverse host responses, aggressive decellularization protocols were frequently performed with a high risk to compromise the natural collagen structure and mechanical properties of the scaffold. None of the* in vitro* studies analyzed have investigated the inflammatory and immunogenic properties of the obtained scaffolds. According to this premise, an interesting approach might be the* in vitro* analyses of inflammatory cytokines (TNF-*α* and interleukins) and cytotoxicity or the immune response in peripheral blood mononuclear cell tests. Only few* in vivo* studies correctly evaluated the inflammatory response by means of histology [20, 21, 25, 30, 36] or immunohistochemistry (B-cells, macrophages) [30], confirming a mild to moderate reaction towards the implanted scaffold within orthotopic [16, 21, 36] but not heterotopic [25, 30] sites. Tischer et al. [16] attributed the partial necrosis and the abundant mononuclear cell infiltration within the fibroblast-reseeded matrix to the ionic detergent (SDS) employed to decellularize the tendon. Kryger et al. [21] demonstrated that acellularized tendons reseeded with various autologous cells behave immunologically like native tendon grafts without the inflammatory response that occurred in allogenic tendons. Omae and colleagues [35, 36] evaluated the gene expression of MMP3 and MMP13 after the* in vitro* reseeding and orthotopic implantation* in vivo*, demonstrating a higher expression in reseeded tendon with respect to the decellularized ones. Nevertheless, the histological analysis did not show any inflammatory cell infiltration in either seeded or unseeded constructs at day 14 after orthotopic implantation.

An important limitation in creating biological vital scaffolds is related to the time necessary to repopulate the decellularized matrix with proper cells, particularly concerning the primary cell lines. Indeed, the primary problem with reseeded matrix is the lag time required for graft preparation prior to implantation starting from the biological material collection through the cell isolation and expansion to the final, functional graft colonization. This temporal limitation is mostly perceived in traumatic acute tissue damage rather than chronic injuries. A cell-based approach for tendon restoration by means of reseeded grafts has been extensively demonstrated as an effective strategy [5]. In this context, an unresolved debate exists on the most effective but less time-consuming cell type for functional tendon graft development to be used in clinics. In fact, among several cell types used to reseed tendons, the most popular are the mesenchymal stem cells (MSCs) associated with long-term culture or cells derived from terminally differentiated tissues (tenocytes, fibroblasts, etc.) with a quicker proliferation rate. Despite the supposed advantages in using high proliferative cells, employing autologous tenocytes or fibroblasts requires invasive harvesting with high patient morbidity without taking advantage of immunomodulatory effects. In addition, the easier harvest of great amounts of mesenchymal stem cells from bone marrow or adipose tissue and approved standard procedures for their isolation better support the use of these cells. Moreover, the immunomodulatory properties of mesenchymal stem cells are well known and exploited in the treatment of therapy-resistant graft-versus-host disease [58]. This peculiar feature of MSCs may play a role in the maintenance of implanted scaffold tolerance and control both the autoimmunity and the inflammatory responses.

Another important issue after the graft reseeding is the viability of seeded cells and their capability in colonizing the scaffold after implantation. Most of the analyzed studies demonstrated a good cell repopulation mainly on the surface of the implanted scaffold after 2 to 8 weeks [21, 22, 28, 32, 36, 44]. Only one author declared a good cellular penetration into the core graft up to 20 weeks after implantation [11].

All vertebrate tendons are relatively similar in elastic modulus (EM), though reported EM varies widely from very low values (160 MPa) to exceeding values (2000 MPa) [59]. Thus, the data interpretation of the analyzed studies becomes difficult due to the different methodologies and measurements performed. The biomechanical properties of decellularized or reseeded tendon matrix need to be maintained similar to the native tissue. Taking into account the basal mechanical properties of human tendons could be a useful benchmark to evaluate the scaffold quality. In the literature, the biomechanical values of human tendons are widely described with a variable EM from 816 ± 218 MPa to 1673 MPa for the Achilles [60] and flexor* digitorum profundus* tendons [61], respectively. Otherwise, the UTS ranges consistently from 40 to 100 MPa [62], or differently reported as 1189 ± 496 N [63]. Aiming at a suitable biological scaffold derived from tendon to tendon substitution in human medicine, xenografts represent promising materials. According to this, most of the studies focused the attention on the protocol optimization in tendons derived from various animals without considering the translatability of the final construct. In particular, decellularizing tendons derived from laboratory [12–24] or small animals [33–39, 57] clearly might not be useful for human use because tissues of these species are not available as commercial products, though they represent a valid approach in research and the results obtained from these studies are still highly relevant. Differently, tendons derived from large animals like pigs [40–44], horses [45–48], bovine [49], or humans [26–32] fit better for this purpose. This concept is particularly true whether considering the final dimensions and biomechanical features of the decellularized or reseeded scaffolds. The studies that processed porcine tendons showed poor biomechanical properties of the native tissue compared to the human basal values [41–44], despite being suitable in terms of dimension. Among the few studies performed on equine tendons, only Youngstrom and colleagues [46, 48] carried out biomechanical tests on native and decellularized tissue, demonstrating inferior properties in terms of EM and failure stress. The analysis of only two similar studies does not allow drawing a firm conclusion on the suitability of equine tendons. Studies performed on the human-derived flexor digital tendons demonstrated that this tissue could be suitable as xenograft in terms of both the EM and UTS falling within the parameters of normal values [27, 28, 30, 31], with the exception of the values reported in the study conducted by Schmitt and colleagues [32]. Some studies working on rabbit tendons [12–14, 17, 18] confirmed that this species has suitable UTS and EM basal values compared to humans. Hence, rabbit tendons are valid candidates to investigate innovative strategies to obtain tendon-derived matrix in the research field, even though they cannot be implantable xenografts.

The decellularization and/or reseeded protocols used to create tendon-derived matrix grafts vary widely throughout the literature, making the comparison of the efficacy of different approaches complex. Indeed, only some* in vitro* studies compared more than two protocols [11, 13, 19, 23, 27, 31, 40, 42, 43, 45, 46], but they differed a lot in terms of starting treatment (fresh or frozen), chemical or enzymatic agents, and concentrations for the decellularization protocols. In addition, the strategy to reseed the matrix is commonly performed by employing different cell sources, types, and days of culture. From these studies emerges the fact that TBP [23, 42] and SDS are the most efficacious reagents to obtain a performing decellularized tendon matrix. An interesting approach to ameliorating the action of chemical detergents in the compact collagen structure of tendons is the use of the peracetic acid (PAA) that acts on the permeability of the matrix permitting a greater penetration of the reagents [25, 27–30, 32]. Thanks to the PAA activity, the total amount and concentration of detergents could be drastically reduced. In fact, the use of harsh decellularization methods might determine damage of the collagen ultrastructure, impairing the biomechanical properties of the decellularized matrix. Conversely, applying a mild decellularization could not fully remove the cell component. Therefore, few studies combined the use of ion and zwitterionic detergents with protease inhibitors such as aprotinin and leupeptin [26, 43] to prevent the matrix disruption by proteases.

An appropriate terminal sterilization of the biological scaffold is mandatory to eliminate endotoxins and bacterial, viral, or prion presence [64], while preserving the structural, biochemical, and mechanical properties of the ECM. According to the literature, a valid sterilization approach is the use of hybrid methods both to sterilize the scaffold and to preserve the biomechanics [65]. Most of the sterilizing techniques use acids (e.g., PAA) or solvents for* in vitro* studies, although they are not sufficient for the clinical translatability as the sterilization required for medical devices needs to achieve sterility insurance levels of 10^−6^ (SAL6) [66]. Other sterilization methods are approved for clinical use, that is, ethylene oxide [39, 57], gamma, and electron beam irradiation, though some authors showed undesirable host immune responses to these processes [67–69]. In the last few years, an alternative and recently approved sterilization method of biological grafts for clinical use is the supercritical carbon dioxide [66, 70]. Due to the intrinsic differences in the ECM composition of various tissues, decellularization agents, time of exposure, and terminal sterilization techniques should be deeply investigated.

### 3.3. Animal Models for Tendon Repair with Decellularized Matrix

Only 12 studies verified the performances of the decellularized constructs unseeded or cell reseeded in animal models. The defect size and surgical techniques used to allocate the grafts are resumed in Table 2. In particular, most of them (67%) described an orthotopic implantation in rabbits [16, 18, 21, 22, 36, 38] and only two studies described an orthotopic implantation in rat Achilles tendon defects [24, 44]. Furthermore, some authors performed subcutaneous implantations of the constructs in mice and rats to investigate both the host inflammatory and the cell-mediated immune responses to detergent remains of decellularization process [25, 28, 29, 32, 44]. In our analysis, we found a high variability of the time points after implantation that were commonly longer in orthotopic models ranging from 2 to 30 weeks (mean 8.4 ± 7.2) than in heterotopic sites, of which the subcutaneous implantation protracted for 1 to 4 weeks (mean 2.7 ± 1.2). Although investigations at different time points are detrimental to evaluate the tendon-tendon integration and the host immune response, the comparison of results among various studies becomes difficult for the aforementioned variability.

#### 3.3.1. Observation on Preclinical Animal Models

In the literature, different species are described as suitable models to study tendon ruptures and tears in terms of the physiopathology and tissue regeneration. Among them, nonhuman primates and dogs were frequently used for this purpose in the past, but the ethical issues and the public opinion decreased their employment in preclinical research [71–73]. Differently, horses, goats, and sheep represent the elective models due to proper dimensions, qualities, and biomechanics of the flexor tendon structure [74–77]. Anyway, the special management, handling, and high costs of these models constitute a limitation for their use. Laboratory animals, like mice and rats, have several advantages as preclinical models. They are very prolific obtaining high numbers for experimental studies; they can be easily handled and are with reduced economic impact. Furthermore, their genome has been completely sequenced showing a high genetic homology to humans (80–90%), thus permitting creating transgenic models of tendinopathy to study its pathogenesis [78]. Nevertheless, their small dimensions, in particular that of mice [79], represent the major limitation to testing novel therapeutic approaches for tendon augmentation and/or laceration. The highest balance in terms of tendon structure, physiology, cellular component, and biomechanics compared to humans can be found in rabbits [80–82]. This species merges appropriate tendon size with an easy handling and controlled costs that make rabbits the most proper animal models to investigate biological tendon graft features before the human use [11–22]. More importantly, the animal models are widely employed to verify the integration of the decellularized matrix within the orthotopic site of implantation. Most of the analyzed studies evaluated the integration of the tissue engineered tendon grafts in a rabbit model of anterior cruciate ligament [16] or tendon defect replacement [18, 21, 22, 36, 38] by means of histological, biomechanical, or molecular investigations. Only one study assessed the graft integration in a rat model of Achilles tendon defect [24]. Overall, these studies reported weaker biomechanical properties after 8–10 weeks of implantation compared to the native tissue [16, 18, 38]. Histologically, a mild to severe inflammatory response was detected after the implantation of reseeded tendon matrix [16, 21, 24, 38]. One group evaluated the expression of tenogenic genes after implantation, reporting higher expression in the reseeded tendon matrix when compared to the decellularized matrix [36].

## 4. Conclusions

The purpose of the present review is to critically analyze the recent literature on tendon-derived biological scaffolds highlighting the principal features to develop a functional tendon device, since the final goal of regenerative medicine and tissue engineering is to translate the tendon restoration to clinics. In particular, we focused on the proper tendon source and dimensions, the safety of the device in terms of host integration, response, and biodegradability related to the common applied protocols for decellularization. Moreover, reseeding and sterilization techniques as well as the adequate mechanical proprieties of the biological product were discussed. More importantly, we evaluated the importance and pertinence of preclinical animal models related to the type and site of tendon injury before evidence based clinical trials.

Biological scaffolds have been increasingly involved in regenerative medicine and tissue engineering because there is no better way to replace a tissue with its homologous structure. Currently, the use of decellularized tendon appears to be an interesting approach for the treatment of tendon ruptures and tears, being the most adequate structure to guide the regeneration of the injured tissue by preserving the complex matrix architecture. The collagenous structure of decellularized tendons is effectively an ideal environment to encourage cell incorporation, metabolism, and matrix synthesis. The purpose of any decellularization protocol is to successfully remove cellular components and nucleic residual, thereby minimizing modifications on the arrangement, biological activity, and mechanical properties of the matrix. Therefore, the ideal tendon graft is a nonimmunogenic, readily acquired ECM with mechanical properties resembling the native tendon features. Up to the present, synthetic biomaterials have not demonstrated appropriate mechanical properties suitable for orthotopic implantation. Conversely, the biological grafts seem to have more appropriate structure and features to be employed for tendon repair. Indeed, xenogeneic tissues, such as dermis, small intestine submucosa, and pericardium, have become popular commercial scaffolds for this purpose as widely demonstrated in the literature [7, 8, 83–86]. Despite the optimal integration, the biocompatibility with the host tissues, and the augmentation function of the commercial biological scaffolds, these products possess low mechanical properties unable to support the physiological loading to be used as tendon substitutes. Moreover, the procedures adopted to decellularize commercial biological scaffolds are commonly protected by an industrial knowhow that impedes the reproducibility and the direct comparison of the protocols.

There are also several limitations in comparing approaches and techniques presented in the literature, even if the aim of their research is the same. In fact, drawing a conclusion is an almost impossible challenge if we consider the use of tendon derived from several species, the different dimensions of the tissue treated with mechanical methods or chemical detergents at different concentrations, and the use of a variety of cell types cultured either in static or in dynamic conditions to recreate implantable constructs tested in different animal models. This potentially raises a concern about the impact of this analysis for further methodology development.

The ultimate animal species—providing tissue for xenotransplantation—should have some characteristics consistent with the clinical use. The limited risks of disease transmission, the appropriate dimensions, and the large availability independent of ethical concern are mandatory. Equine tendon xenografts could meet the requirements thanks to an inferior and geographically limited zoonosis, proper dimension, and biomechanical characteristics similar to human tendons and being the tissue harvest related to slaughter waste products. Moreover, the development of a tissue substitute derived from equine might also be employed in the reconstruction of tendon injuries commonly occurring in athletic horses or pets, thus creating a commercial product that can be also sold in the veterinary market. As emerged from our review, only few studies investigated equine tendon as source and none of them appears to demonstrate an adequate DNA removal. Thus, further evidences are required to support the use of this species.

As emerged from our analysis, efficient decellularization protocols require a combination of physical, enzymatic, and chemical treatments to eliminate as much of cellular residues as possible to make the construct safe for transplantation. From these studies emerges the fact that TBP and SDS are the most efficacious reagents to obtain a performing decellularized tendon matrix, while preserving the mechanical properties of the native tendon. An interesting approach to ameliorate the final product characteristics is the association of these reagents with endonucleases (RNAse or DNAse) in order to avoid immunogenicity caused by cell residues and the use of protease inhibitors (aprotinin and leupeptin) to prevent matrix disruption or weakening by proteases. Also the removal of reagent remaining is mandatory to contain the inflammatory response; hence supplementary investigations could be done to verify the construct biocompatibility such as the evaluation of cytotoxicity or proinflammatory cytokines release.

The ECM structure might become more compact or more inhomogeneous in response to decellularization treatments; thus the use of detergents like PAA could promote cell penetration and migration through the matrix improving scaffold permeability. PAA is also frequently used as a sterilizing detergent even though it is not an approved method for the clinical translatability. From a clinical point of view, it will be important to further investigate a sterilization method able to achieve sterility insurance levels without compromising biomechanical and biocompatible features of the scaffold.

The biological scaffolds derived from decellularized tendon are often reseeded with different cell types to recreate implantable constructs either in static or in dynamic culture conditions. Tenocytes, fibroblasts, bone-marrow- (BMSCs), adipose- (ASCs), and tendon-derived (TSPCs) stromal cells are all possible candidates for tissue engineering approaches. Despite that, the use of BMSCs is encouraged because of their advantageous characteristics, requiring less invasive harvesting procedures and their immunomodulatory features being favorable for transplantation with respect to cells isolated from other tissues. As the goal of decellularizing tendons apparently is to reseed them with host cells before implantation, one of the most challenging aspects is optimizing the cell invasion of such a dense tissue like tendon. Thus, the use of bioreactors supports the cell penetration and distribution within the matrix structure to accelerate the* in vitro* production of biological constructs able to better support the host integration and functionality after implantation while maintaining viable the reseeded cells.

The obtained implantable tendon devices have been successfully implanted in preclinical animal models to reconstruct experimentally induced tendon damage or tissue loss. In the literature, different species are described as proper models to study the physiopathology and regeneration pathway of this tissue, but the highest balance compared to humans' features can be found in rabbits, merging appropriate tendon size with contained costs and an easy handling. However, the best choice of animal model depends on the research question and expected results in terms of tendon rupture and repair and scaffold integration.

In conclusion, the detection of a proper extracellular matrix as a tissue substitute able to deliver viable autologous cells and eventually biological agents might be a promising approach in the regeneration of injured tendon. However, it will be critical to standardize decellularization or reseeding protocols to obtain reproducible products able to become a near-term clinical reality.

## Figures and Tables

**Table 1:** *In vitro* studies of tendon tissue decellularization.

Tendon source/size	Decellularization protocol(s)	Assessment of decellularization	Cell reseeding	Reseeding assessment	Experimental groups	Results and comments	Reference
Rabbit flexor FDP 3–5 cm length	Frozen −80°C (until use) 0.05% Trypsin/EDTA 37°C (24 h) 0.5% Triton X100 RT (24 h) 0.25% Trypsin 37°C (15′) 0.5% Collagenase 37°C (1 h)	Histology Cell count	Rabbit tenocytes 2 × 10^6^ cells/mL Static culture for 4 days	Mechanical testing	Freeze-stored FDP tendon; acellularized FDP matrix; reseeded FDP matrix; fresh native FDP tendon (ctrl)	Acellularized matrix → absence of cells or morphological alteration; UTS/EM similar to ctrl Reseeding matrix → cells present only on the surface; EM similar to ctrl	[12]

Rabbit ST and FDP 2 cm length	Stored in 0.02% EDTA 4°C (until use) *Protocol 1*: 1% Triton X100 (24 h) *Protocol 2*: 0.5% SDS (24 h) *Protocol 3*: 1% TBP (24 h) *Protocol 4*: 1% Triton X100 + 0.5% SDS (24 h) *Protocol 5*: 1% TBP + 0.5% SDS (24 h) *Protocol 6*: 1% TBP + 1% Triton X100 (24 h) Followed by RNAse/DNAse 37°C (24 h) 0.02% EDTA 4°C (24 h)	Histology Mechanical testing	—	—	Acellularized ST/FDP matrix; fresh native ST/FDP tendon (ctrl)	*Protocol 4* → collagen ruptures *Protocol 5* → no impact on tendon structure and collagen content Acellularized matrix → histology and biomechanics similar to ctrl	[13]

Rabbit FDP 5 cm length	Frozen −80°C (until use) 0.05% Trypsin/EDTA 37°C (24 h) 0.5% Triton X100 RT (24 h)	Histology Cell count	Rabbit tenocytes 2 × 10^6^ cells/mL Rotating reseeding (24 h) Static or dynamic culture (LigaGen L30-4C) for 5 days	Histology Mechanical testing	Freeze-stored acellularized FDP matrix (AC); acellularized FDP matrix + bioreactor loading (A+); acellularized FDP matrix w/o bioreactor loading (A−); reseeded FDP matrix + bioreactor loading (R+); reseeded FDP matrix w/o bioreactor loading (R−); fresh native FDP tendon (NC); freeze-stored FDP tendon (FC)	NC → highest UTS/EM R+ → higher UTS/EM compared to FC, AC, R−, and A−	[14]

Rabbit ST Undefined size	Frozen −80°C (until use) 1% SDS (24 h)	—	Rabbit dermal fibroblasts 2 × 10^6^ cells/mL Static culture by injection for 4, 7, and 14 days	Histology Mechanical testing Immunohistochemistry	Acellularized ST matrix; reseeded ST matrix; fresh native ST tendon (ctrl)	Acellularized matrix → histology and immunohistochemistry similar to ctrl Procollagen I → present in ctrl and reseeded matrix, absent in acellularized matrix Collagens I-III-IV-VI, versican → similar among treatments UTS/stiffness/elongation → higher in reseeded matrix	[15]

Rabbit FDP 5 cm length	Frozen −80°C (until use) 0.05% Trypsin/EDTA 37°C (24 h) 0.5% Triton X100 RT (24 h)	Histology Cell count	Rabbit ASCs and Fs 2 × 10^6^ cells/mL Rotating reseeding (24 h) Static or dynamic culture (LigaGen L30-4C) for 5 days	Histology Mechanical testing	Reseeded FDP matrix + bioreactor loading (ASCs+); reseeded FDP matrix w/o bioreactor loading (ASCs−); reseeded FDP matrix + bioreactor loading (Fs+); reseeded FDP matrix w/o bioreactor loading (Fs−); fresh native FDP tendon (ctrl)	UTS/EM → higher in ASCs+, Fs+ versus ASCs−, Fs−, ctrl ASCs+, and Fs+ → cells oriented parallel to the direction of strain	[17]

Rabbit FDP 1.5 cm length	Fresh samples *Protocol 1*: 0.5% SDS (30′) *Protocol 2*: 0.05% Trypsin/EDTA (24, 48, and 72 h) *Protocol 3*: 0.05% Trypsin/EDTA (24 h); 0.5% Triton X100 (24 h) *Protocol 4*: 3% Triton X100 (24, 48 h) *Protocol 5*: 3% Triton X100 (24 h); 0.5% SDS (24 h) *Protocol 6*: Frozen −70°C (until use); 0.05% Trypsin/EDTA (24 h); 0.5% Triton X100 (24 h)	—	Rabbit tenocytes (endotenon, epitenon) 1 × 10^6^ cells/1.5 cm construct Static culture for 1, 3, and 6 weeks	Histology Cell fluorescent labeling	Reseeded FDP matrix with endotenon cells; with epitenon cells; and with mixed cells	Protocol 6 → best decellularization process Epitenon/endotenon cells → attached to the periphery of the acellularized matrix Endotenon cells → penetrated the matrix core by 3 weeks	[11]

Rabbit PT 3 cm length; 1.5 cm width; 3 mm thickness	Frozen −20°C (until use) *Protocol 1*: 1% TBP RT (48, 72 h) *Protocol 2*: 1% SDS RT (24, 48 h)	Histology GAG content Mechanical testing	Human HS68 2 × 10^5^ cells/mL Static culture for 4 h, 1, 3, 6, and 14 days	Histology Cell count	Reseeded TBP-treated PT matrix; reseeded SDS-treated PT matrix; fresh native PT (ctrl)	SDS and TBP → 70–90% of cell removal Reseeded matrix biomechanics → similar to ctrl TBP-treated matrix → supported cell proliferation better than SDS-treated matrix and was histologically similar to ctrl	[19]

Rabbit PT 1.5 cm length; 0.3 cm width; 1 mm thickness	Frozen liquid nitrogen/thaw (5 cycles) Frozen −80°C (until use)	—	Rabbit tendon fibroblasts 5 × 10^6^ cells/mL Static culture in collagen gel with or w/o anti-TGF*β*1 antibody for 6 weeks	Mechanical testing Cell fluorescent labeling	Acellularized PT matrix; reseeded PT matrix; reseeded PT matrix + anti-TGF*β*1 antibody	Anti-TGF*β*1 antibody decreased UTS/EM in reseeded matrix	[20]

Canine FDP 2 cm length	Frozen −80°C (until use) 0.05% Trypsin/EDTA 37°C (24 h) 0.5% Triton X100 RT (24 h)	Mechanical testing	Canine BMSCs 2 × 10^7^ cells/mL Static culture for 2 weeks	Histology Cell/DNA content LiveDead	Acellularized FDP matrix; acellularized FDP matrix, perforated with multiple slits (MS); acellularized FDP matrix, perforated with multiple slits + hyaluronic acid gelatin (MS-SM); reseeded FDP/MS matrix; fresh native FDP tendon (ctrl)	UTS → higher in acellularized FDP/MS matrix compared to ctrl DNA content → lower in MS than ctrl; higher than in acellularized matrix with or w/o BMSCs BMSCs were viable after 2 weeks	[34]

Canine FDP Undefined size	Frozen −80°C (until use) 0.05% Trypsin/EDTA 37°C (24 h) 0.5% Triton X100 RT (24 h)	Histology SEM Mechanical testing	—	—	Acellularized FDP matrix; acellularized FDP matrix + hyaluronic acid gelatin (GT); fresh native FDP tendon (ctrl)	UTS → higher in acellularized matrix than ctrl and GT EM → similar among treatments Matrix surfaces → smooth in ctrl and GT; rough in acellularized matrix	[33]

Canine AT 4 cm length; 0.3 mm thickness	Frozen −80°C/thaw (5 cycles) RNAse/DNAse 37°C (4, 8, and 12 h)	Histology Cell/DNA content SEM Mechanical testing Biochemical analysis	Murine fibroblasts (3T3) 1 × 10^6^ cells/mL Static culture for 4 days	Histology SEM	Acellularized AT matrix (only freeze/thaw); acellularized AT matrix (freeze/thaw + nuclease treatments); fresh native AT (ctrl)	Complete cell removal → in repetitive freeze/thaw + nuclease treatment for 12 h Acellularized matrix → ultrastructure, proteoglycans, and growth factors were preserved; UTS was retained in 85.62% of ctrl	[37]

Canine IT 2.5 cm length; 1 cm width; 0.05 mm thickness	Frozen −80°C (until use) Frozen liquid nitrogen/thaw (5 cycles) RNAse/DNAse 37°C (12 h)	—	Canine BMSCs 5 × 10^6^ cells/mL Static culture for 2, 7, and 14 days	Histology Cell fluorescent labeling Gene expression Mechanical testing	Acellularized IT matrix; reseeded IT matrix; fresh native IT (ctrl)	Reseeded matrix → viable cells aligned to the collagen fibers; higher tenomodulin, MMP13 and lower collagen I than in BMSCs before seeding Biomechanics → similar between acellularized and reseeded matrixes	[35]

Canine AT 4 cm length; 0.3 mm thickness	Frozen liquid nitrogen/thaw (5 cycles) RNAse/DNAse 37°C (12 h)	SEM Mechanical testing	Rat BMSCs and TSPCs 2 × 10^5^ cells/cm^2^ Static culture for 6 h and 1, 3 days	LiveDead Cell proliferation SEM	Acellularized AT matrix; reseeded AT matrix; fresh native AT (ctrl)	Acellularized matrix → preserved ECM and EM similar to ctrl Reseeded matrix → homogenous cell distribution, alignment, and tenogenic differentiation of seeded cells	[57]

Canine AT 4 cm length; 0.3 mm thickness	Frozen liquid nitrogen/thaw (5 cycles) RNAse/DNAse 37°C (12 h)	—	Canine BMSCs 5 × 10^5^ cells/mL Dynamic culture for 1, 3, and 7 days	Histology Cell fluorescent labeling SEM Mechanical testing	Acellularized AT matrix; reseeded AT matrix; fresh native AT (ctrl)	Reseeded matrix → homogenous cell distribution at day 7; tenogenic differentiation of BMSCs; UTS similar to ctrl	[39]

Porcine AT 1 cm length; 0.5 cm width; 3 mm thickness	Frozen −20°C (until use) *Protocol 1*: 3% Triton X100 37°C (24 h) *Protocol 2*: 1% SDS + 0.2% natrium acid + 5 mM EDTA RT (24 h) *Protocol 3*: 0.05% Trypsin/EDTA RT (24 h)	—	Human tenocytes 4 × 10^6^ cells/cm^2^ Centrifuging reseeding + static culture Rotating culture Reseeding by injection + rotating culture for 1, 3 weeks	Histology Cell fluorescent labeling DNA/GAGs content	Acellularized porcine AT matrix; reseeded porcine AT matrix; fresh native human/porcine AT (ctrl)	Porcine ctrl → higher cell/GAGs content; more compact and wavy pattern than human ctrl Acellularized matrix → lower GAGs/DNA content Reseeded porcine matrix → greater cell/GAGs content compared to ctrl; inhomogeneous, superficial, and not aligned tenocyte distribution Rotating culture → greater cell/GAGs content compared to centrifuging-based seeding, lower cell/GAGs content compared to ctrl Cell injection → poor cell distribution; no GAGs deposition	[40]

Porcine ATT 12 cm length; 1 cm width	Frozen −70°C (until use) 0.25% Trypsin/collagenase type I 37°C (4 h) NaCl washing 4°C (3 cycles) (1 h) Ultrasonication (5′)	Histology DNA/GAG/collagen SEM Mechanical testing	Dynamic culture w/o cells in bioreactor (110% tension + 90° torsion) for 1, 3, and 7 days	—	Acellularized ATT matrix; acellularized ATT matrix cultured in bioreactor; fresh native ATT (ctrl)	Acellularized matrix → lower DNA content; lower UTS compared to ctrl Acellularized matrix treated in bioreactor → greater UTS than ctrl	[41]

Porcine DT Undefined size	Stored at 4°C (until use) *Protocol 1*: 0.1% PAA + 4% EtOH stirring RT (16 h) *Protocol 2*: 1% Triton X100 RT (24 h) *Protocol 3*: 1% Triton X100 + 1% TBP RT (24 h) *Protocol 4*: 2% TBP RT (24 h) *Protocol 5*: 1% TBP RT (24 h) *Protocol 6*: 1% SDS RT (24 h) *Protocol 7*: 0.5% SDS RT (24 h)	Histology Cell viability SEM Mechanical testing Hydroxyproline	—	—	Acellularized DT matrix; stored native DT (ctrl)	*Protocol 5* → good cell removal; tissue structure and composition were preserved Acellularized matrix → UTS, EM, and hydroxyproline release were similar to ctrl	[42]

Porcine PT 6 cm length; 1.5 cm width; 5 mm thickness	*Protocol 1*: 0.1% EDTA + aprotinin RT (24 h); 0.1% SDS RT (24 h); RNAse/DNAse 37°C (3 h) *Protocol 2*: 0.1% EDTA + aprotinin RT (24 h); 0.1% SDS RT (24 h); RNAse/DNAse 37°C (3 h)	Histology Immunohistochemistry Hydroxyproline Collagen/GAGs Mechanical testing	Human tenocytes 1 × 10^5^ cells/mL Static culture for 3 weeks	Histology LiveDead	Acellularized PT matrix; acellularized + sonicated PT matrix; reseeded PT matrix; reseeded + sonicated PT matrix; fresh native PT (ctrl)	Ultrasonication treatment → optimal treatment; collagen and GAGs were similar to ctrl Reseeded + sonicated PT matrix → centrally colonized by unviable cells	[43]

Human FDP 2 cm length; 0.25 cm width	0.1% EDTA + protease inhibitor 37°C (24 h) 0.1% SDS 37°C (5 h) DNAse RT (1 h)	—	Human ASCs 1 × 10^6^ cells/0.5 mL Static culture by injection for 7 days	Histology Cell/DNA content Immunohistochemistry	Acellularized FDP matrix; reseeded FDP matrix; reseeded FDP matrix + collagen solution; fresh native FDP tendon	Acellularized matrix → complete cell removal, absence of DNA content, and no changes in ECM structure Reseeded matrix → cell distribution on surface Reseeded matrix + collagen → deeper penetration of collagen, COMP positive, and viable cells aligned to collagen fibers	[26]

Human FDP 1.5 cm length	Frozen −70°C (until use) *Protocol 1*: 0.1% EDTA RT (4 h); 0.1% SDS + 0.1% EDTA RT (24 h) *Protocol 2*: 0.1% EDTA RT (4 h); 0.1% SDS + 0.1% EDTA RT (24 h); 2, 5, and 10% PAA (4, 20 h)	Histology	Human dermal fibroblasts 1 × 10^6^ cells/mL Rotating culture for 5 days	Cell/GAGs/collagen content LiveDead Mechanical testing	Acellularized FDP matrix with 5% PAA; reseeded FDP matrix	Protocol 2 → increased porosity, improved cell penetration, and migration Acellularized matrix → similar collagen/GAG content when treated with or w/o PAA; UTS/EM lower than in reseeded matrix	[27]

Human FDP 1 cm length	0.1% SDS + 0.1% EDTA RT (24 h) 5% PAA (6 h) frozen −80°C (until use)	—	Human fibroblasts, tenocytes, and ASCs 5 × 10^5^ cells/mL Rotating culture with or w/o growth factors for 5 days	Histology Cell count Immunohistochemistry	Acellularized FDP matrix; reseeded FDP matrix; fresh native FDP tendon (ctrl)	Enhanced cell proliferation using 5 ng/mL bFGF, 50 ng/mL IGF-1, and 50 ng/mL PDGF-BB Reseeded matrix + growth factors → improved reseeding compared to ctrl w/o growth factors	[29]

Human FDP 1 cm length	Frozen −70°C (until use) 0.1% EDTA (4 h) *Protocol 1*: 1% Triton X100 (24 h) *Protocol 2*: 1% TBP (24 h) *Protocol 3*: 1% SDS (24 h) *Protocol 4*: 0.1% SDS (24 h)	Histology DNA/GAGs/collagen Mechanical testing	Human dermal fibroblasts 0.5, 1, 2 × 10^6^ cells/mL Static culture for 6, 24 h	Histology Cell count LiveDead	Acellularized FDP matrix; reseeded FDP matrix; fresh native FDP tendon (ctrl)	*Protocols3*/*4* → lower DNA content compared to ctrl Acellularized matrix → good collagen/GAG content UTS was similar to ctrl (especially *Protocol 4*) Reseeded matrix → cell distribution on surface	[31]

Equine SDFT and DDFT 3 cm length; 1 cm width; 2 mm thickness	*Protocol 1*: frozen liquid nitrogen/thaw (5 cycles); 1% Triton X100 RT (48 h) *Protocol 2*: frozen liquid nitrogen/thaw (5 cycles); 1% SDS RT (48 h) *Protocol 3*: 1% Triton X100 RT (48 h) *Protocol 4*: 1% SDS RT (48 h)	Histology Cell/DNA content TEM	Equine ASCs 1.3 × 10^5^ cells/cm^2^ Static culture for 7, 14 days	Histology MRI LiveDead	Acellularized SDFT/DDFT matrix; reseeded SDFT/DDFT matrix; fresh native SDFT/DDFT (ctrl)	Acellularized matrix → lower DNA content in *Protocols 1*/*2* (1% residual nuclei; 20% residual DNA) compared to *Protocols3*/*4* (20% residual nuclei; 40% residual DNA); absence of morphological ECM alterations Reseeded matrix → best cell distribution in *Protocol 1*	[45]

Equine SDFT 4 cm length; 1 cm width; 0.4 mm thickness	Frozen −80°C (until use) *Protocol 1*: 1% TBP 4°C (48 h) *Protocol 2*: 1% SDS + 0.5% Triton X100 4°C (48 h) *Protocol 3*: 1% SDS 4°C (48 h) *Protocol 4*: 2% SDS 4°C (48 h) Followed by 0.05% Trypsin/EDTA (10′) DNAse (30′)	Histology DNA/GAGs/collagen LiveDead SEM Mechanical testing	Equine BMSCs 2 × 10^4^ cells/cm^2^ Static culture for 11 days	Histology Cell count LiveDead	Acellularized SDFT matrix; reseeded SDFT matrix; fresh native SDFT (ctrl)	*Protocols2*, *3*, and *4* → lower DNA/GAGs content compared to ctrl (especially *Protocol 4*); preserved collagen content; UTS/EM were similar to ctrl Reseeded matrix → higher cell proliferation/integration in *Protocol 4*	[46]

Equine SDFT 4.5 cm length; 0.4 mm thickness	Frozen/thaw (4 cycles) 2% SDS 4°C (48 h) 0.05% Trypsin/EDTA (10′) DNAse (30′)	Histology DNA/GAGs/collagen Mechanical testing	Equine BMSCs 2 × 10^4^ cells/cm^2^ Static and dynamic culture for 11 days	Histology DNA/GAGs/collagen Mechanical testing	Acellularized SDFT matrix; reseeded SDFT matrix; fresh native SDFT (ctrl)	Acellularized matrix → GAGs lost Reseeded matrix → best dynamic protocol for BMSCs integration and tenogenic differentiation was cyclic strain of 3% at 0.33 Hz; UTS/EM greater than acellularized matrix; GAGs similar to ctrl	[48]

Equine SDFT 1 cm length; 1 cm width; 0.5 mm thickness	Frozen −80°C/thaw (4 cycles)	—	Equine BMSCs and TSPCs 1.25 × 10^5^ cells/cm^2^ Static culture for 7 days	Histology Cell/GAGs/collagen Gene expression	Acellularized SDFT matrix; reseeded SDFT matrix (BMSCs); reseeded SDFT matrix (BMSCs + IGF1); reseeded SDFT matrix (TSPCs); reseeded SDFT matrix (TSPCs + IGF1)	Acellularized matrix → cell numbers 1.6- to 2.8-fold higher for TSPCs than for BMSCs and 0.8- to 1.7-fold higher for IGF-I-treated than for untreated cells TSPCs IGF1-treated cell → greatest collagen/GAGs content Collagens I, III, COMP → similar among groups	[47]

Bovine AT 2 cm length; 2 cm width; 2 mm thickness	Frozen (until use) 1% SDS 4°C (48 h) 0.1 mM EDTA (48 h)	SEM Mechanical testing	—	—	Acellularized matrix; acellularized matrix crosslinked with 0.1, 0.5, 1, and 2.5% of glutaraldehyde	Crosslinked acellularized matrix → UTS/EM greater than acellularized matrix	[49]

Rat tail tendon 2-3 mm Ø	Frozen −20°C (until use) *Protocol 1*: 1% Triton X100 (24 h) *Protocol 2*: 1% Triton X100 + 1% TBP (24 h) *Protocol 3*: 1% TBP (12, 24, and 48 h) *Protocol 4*: 2% TBP (24 h) *Protocol 5*: 0.5% SDS (24 h) *Protocol 6*: 1% SDS (12, 24 h)	Histology Mechanical testing	—	—	Acellularized tail tendon matrix; fresh native rat tail tendon (ctrl)	*Protocol 1* → collagen fiber disruption; no cell removal *Protocol 3* (48 h) or *Protocol 6* (24 h) → good acellularized matrix, normal structure and mechanical properties	[23]

FDP = *flexor digitorum profundus*; ST = *semitendinosus* tendon; PT = patellar tendon; AT = Achilles tendon; IT = *infraspinatus* tendon; ATT = *anterior tibialis* tendon; DT = diaphragm tendon; SDFT = superficial digital flexor tendon; DDFT = deep digital flexor tendon; TBP = Tributyl Phosphate; SDS = Sodium Dodecyl Sulphate; PAA = peracetic acid; ASCs = adipose-derived stromal cells; Fs = sheath fibroblast; HS68 = human neonatal dermal fibroblast; BMSCs = bone-marrow-derived stromal cells; TSPCs = tendon-derived stromal cells; UTS = ultimate tensile strength; EM = elastic modulus; SEM = scanning electron microscope; TEM = transmission electron microscope; GAGs = glycosaminoglycans; TGF-*β*1 = transforming growth factor-*β*1; IGF-1 = insulin-like growth factor-1; MMP = matrix metalloproteinase; ECM = extracellular matrix; COMP = cartilage oligomeric matrix protein; bFGF = basic fibroblast growth factor; PDGF-BB = platelet-derived growth factor-BB; RT = room temperature; EtOH = ethanol; ctrl = control; w/o = without.

**Table 2:** *In vivo* studies of tendon tissue decellularization and implantation.

Tendon source and size	Decellularization protocol(s)	Assessment of decellularization	Cell reseeding	Animal model	Implant site, size, and time point(s)	Experimental groups	Assessment of tendon repair	Results and comments	Reference
Rabbit ST Undefined size	Frozen −80°C (until use) 1% SDS RT (24 h)	—	Rabbit dermal fibroblasts 2 × 10^6^ cells/mL Static culture by injection for 4 days	Rabbit	ACL replacement 8 weeks	Autologous ST tendon; reseeded allogenic ST matrix; fresh rabbit ACL in knees (ctrl)	Histology Mechanical testing	Before implantation Stiffness → similar in autologous tendon and reseeded matrix EM → higher in reseeded matrix than autologous tendon After implantation Reseeded matrix → weaker biomechanics than autologous tendons; necrosis and tissue remodeling higher than autologous tendon; inflammatory reaction; inhomogeneous cell repopulation	[16]

Rabbit FDP 3 cm length	Frozen −80°C (until use) 0.05% Trypsin-EDTA RT (24 h) 0.5% Triton X100 RT (24 h)	—	Rabbit tenocytes (male) 2 × 10^6^ cells/mL Rotating culture for 24 h	Rabbit (female)	FDP replacement 3, 6, 12, and 30 weeks	Acellularized FDP matrix; reseeded FDP matrix; fresh native FDP tendon (ctrl)	Histology Fluorescent *in situ* hybridization (donor male tenocytes ≠ female tenocytes)	Reseeded matrix → good cell repopulation; similar to ctrl 6 weeks after implantation	[22]

Rabbit FDP 2 cm length	Frozen −70°C (until use) 0.05% Trypsin/EDTA 37°C (24 h) 0.5% Triton X100 RT (24 h)	Histology	Rabbit tenocytes, Fs, BMSCs, and ASCs 2 × 10^6^ cells/mL Static culture for 1, 4, and 8 weeks	Rabbit	FDP replacement 4, 6, and 8 weeks	Autologous FDP tendon (ctrl); allogeneic FDP tendon; acellularized FDP matrix; reseeded FDP matrix	Histology	Reseeded matrix → viable cell distribution on surface, but not into the centre at 1, 4, and 8 weeks, good collagen architecture Autologous, allogenic, and acellularized implants → mild inflammatory response	[21]

Rabbit FDP 2–2.5 cm length	Frozen −70°C (until use) 0.05% Trypsin/EDTA 37°C (24 h) 0.5% Triton X100 RT (24 h)	—	Rabbit tenocytes, ASCs 2 × 10^6^ cells/mL Static culture	Rabbit	FDP replacement 2, 4, 10, and 20 weeks	Autologous FDP tendon (ctrl); acellularized FDP matrix; reseeded FDP matrix	Histology Mechanical testing	Reseeded matrix → UTS similar to ctrl up to 4 weeks; weaker compared to ctrl at 10 weeks; greater in tendon reseeded matrix from 2 to 20 weeks; similar to acellularized matrix at 2, 4, and 20 weeks; cell penetration to the core of the grafts	[18]

Canine AT 4 cm length; 0.4 cm width; 0.3 mm thickness	Frozen −80°C/thaw (5 cycles) RNAse/DNAse 37°C (12 h)	—	—	Rabbit	IT full-thickness defect 8 mm length 4, 8, and 12 weeks	Acellularized AT matrix; void defect	Histology Mechanical testing MRI	Acellularized matrix → host cell ingrowth and tissue integration; developing of a tendon-like structure at 12 weeks; UTS similar to void defect at 4, 12 weeks; stiffness greater at 12 weeks than at 4, 8 weeks and compared to void defect at 12 weeks MRI signal → similar in acellularized matrix and contralateral native tendon at 12 weeks; hypertrophy and scar tissue in void defects	[38]

Canine IT 1 cm length; 1 cm width; 0.05 mm thickness	Frozen −80°C (until use) Frozen liquid nitrogen/thaw (5 cycles) RNAse 37°C (12 h)	—	Rabbit BMSCs 5 × 10^6^ cells/mL Static culture for 24 h	Rabbit	Patellar tendon defect 10 mm length; 3 mm width 2 weeks	Reseeded IT matrix; void defect	Histology Gene expression Cell fluorescent labeling	Reseeded matrix → cells aligned to fibrils before implantation; scattered after implantation; higher tenomodulin, collagen III, MMP3-13, and lower collagen I expressions compared to acellularized matrix after implantation; viable cells expressing tendon phenotype *in vivo*	[36]

Rat AT Undefined	Frozen −80°C (until use) 0.05% Trypsin/EDTA 37°C (24 h) 0.5% Triton X100 RT (24 h)	Histology	Rat tenocytes 1 × 10^6^ cells/mL Static culture for 48 h	Rat	Achilles tendon defect Mid 1/3 portion	Autologous AT (ctrl); acellularized AT matrix; reseeded AT matrix	Histology Mechanical testing	Reseeded matrix → preserved collagen at 12 weeks; best histological score and biomechanics at 24 weeks and better organized ECM compared to ctrl and acellularized matrix Acellularized matrix → less inflammatory cells than ctrl and reseeded matrix	[24]

Human FDP; FDS; FPL 8–10 cm length	Frozen −70°C (until use) 0.1% EDTA RT (4 h) 0.1% SDS + 0.1% EDTA RT (24 h) 5% PAA (4 h)	Histology Cell/DNA content Immunohistochemistry TEM Mechanical testing	Human ASCs-luc2-eGFP 1 × 10^6^ cells/mL Rotating seeding (6 h) Static culture for 2 weeks	Athymic rat	Subcutaneous pocket 3 cm length 4 weeks	Surface scored matrix (S); rehydrated matrix (F); surface scored + rehydrated matrix (S + F); fresh human tendons (ctrl)	Cell fluorescent labeling	Reseeded S and S + F → greater viable cell attachment and penetration compared to reseeded F and ctrl; reduced apoptosis, persistent procollagen production, and similar ultrastructure to ctrl Rehydration of F and S + F did not improve reseeding UTS/EM → greater in reseeded matrix than acellularized matrix	[28]

Human FDP 5 cm length	Frozen −70°C (until use) 0.1% EDTA + 0.1% SDS RT (24 h) 5% PAA (6 h)	Immunohistochemistry	—	Rat	Subcutaneous pocket 2, 4 weeks	Acellularized FDP matrix; fresh native FDP tendon (ctrl)	Histology Immunohistochemistry Mechanical testing	Acellularized matrix → cell and MHC-1 complex removal; preserved collagen structure and increased porosity; UTS/EM greater compared to ctrl; lower inflammatory response and abnormal collagen architecture compared to ctrl B-cell infiltration → detected in ctrl compared to acellularized matrix after implantation Macrophage infiltration → absent after implantation	[30]

Human FDP 4 cm length	Frozen −70°C (until use) 0.1% EDTA (4 h) 0.1% SDS + 0.1 EDTA (24 h) 5% PAA (4 h) Manually scored Dehydrated	Histology DNA content	Human ASCs-luc2 1-2 × 10^6^ cells/mL Static culture for 30 h	Rat	Subcutaneous pocket 7, 14, 21, and 28 days	Acellularized FDP matrix; reseeded FDP matrix	Histology Immunohistochemistry Cell fluorescent labeling Mechanical testing	Reseeded matrix → viable cells during implantation up to 4 weeks Host cell invasion, proliferation, and vascularization were observed after implantation UTS → similar between acellularized and reseeded matrixes Collagen I > III → consistent in reseeded matrix after implantation	[32]

Porcine tendon 1 cm length; 1 cm width; 0.08 mm thickness	Frozen liquid nitrogen/thaw (5 cycles) DNAse 4°C (14 h)	Histology Cell/DNA/collagen SEM Gene expression	Human TSPCs 11.5 × 10^5^ cells/cm^2^ Static culture for 1, 7 days	Athymic mouse Rat	Subcutaneous pocket 4 weeks AT defect (6 mm length) 2, 4 weeks	Acellularized matrix; reseeded matrix	Histology Collagen content TEM Mechanical testing	Reseeded matrix → good cell adhesion and proliferation, promoting a tendon phenotype (scleraxis); mature structure, larger collagen fibrils, and stronger mechanical properties compared to acellularized matrix after implantation No osteogenic behavior was observed in reseeded matrix after implantation	[44]

Chicken FDP 6 cm length; 0.3 cm width; 1 mm thickness	Stored 4°C (until use) 0.05% Trypsin/EDTA 37°C (1 h) 2% Triton X100 + 1.5% PAA 37°C (4 h)	Histology SEM DNA content Mechanical testing	*—*	Mouse	Subcutaneous pocket 2 mm length; 2 mm width; 1 mm thickness 3, 7, 14, and 21 days	Acellularized FDP matrix; fresh native FDP tendon (ctrl)	Histology	Acellularized matrix → decreased or absent DNA content compared to ctrl; UTS/EM 76–78% of that observed in ctrl No host-cell-mediated foreign-body immune response was observed after implantation	[25]

FDS = *flexor digitorum superficialis*; FPL = *flexor pollicis longus*; GFP = green fluorescent protein; luc2-GFP = luciferase 2-green fluorescent protein; ACL = anterior cruciate ligament; MRI = magnetic resonance imaging; MHC-1 = major histocompatibility complex-1.
